# Protease nexin 1: a novel regulator of prostate cancer cell growth and neo-angiogenesis

**DOI:** 10.18632/oncotarget.824

**Published:** 2013-01-27

**Authors:** Chad M. McKee, Danmei Xu, Ruth J. Muschel

**Affiliations:** Gray Institute of Radiation Oncology and Biology, University of Oxford, Oxford, UK; Department of Haematology, Tongji Hospital and Medical College, Huazhong University of Science and Technology, Hubei, China; Gray Institute of Radiation Oncology and Biology, University of Oxford, Oxford, UK

Prostate adenocarcinoma (CaP) is the most frequent cancer and second leading cause of cancer death among men in the Western world [1]. Hh signaling may augment the development of CaP as emerging data suggests that components of the pathway are potential indicators of worse prognosis [2]. One major clinical difficulty, however, is that many prostate cancers are indolent and cause no difficulty while others are aggressive and lead to morbidity and mortality. Current methods to distinguish CaP types are inadequate and could lead to overtreatment in many cases. We recently have shown that genetic alteration in the Hedgehog (Hh) pathway impacts CaP growth experimentally and may correlate with clinical outcome [3]. Examination of a cohort of intermediate risk prostate carcinoma patients by comparative genomic hybridization (CGH) for a panel of hedgehog related genes indicated a more rapid progression to PSA (prostate antigen) failure, bolstering this argument. Thus, studying the pathway may reveal promising avenues for distinguishing outcomes and identifying new targets for therapy.

Our work has shown that Hh signaling can be regulated in part by a protein normally expressed in the prostate, protease nexin 1 (PN1, also known as serpinE2) [3]. PN1 is a serine protease inhibitor (serpin) with the ability to bind and neutralize the activity of thrombin, trypsin, and urokinase (uPA) [4]. The regulation of uPA is meaningful in cancer because of its role in the cleavage and activation of plasminogen to plasmin, which in turn activates matrix metalloproteinases (MMPs) and ECM remodelling [5]. Previously, we established that PN1-mediated inhibition of uPA served to inhibit metastatic prostate cell invasion in Matrigel [6].

Our new study features several components. Firstly, we show that PN1 inhibits Hh signaling by reduction in the hedgehog ligand, Sonic (SHH). This regulation is dependent upon uptake of PN1 through the LRP receptor and on the inhibitory activity of PN1. The immediate functional impact of PN1 is to reduce proliferation of prostate metastatic cells both in culture and *in vivo*. Indeed, inducing expression of PN1 in several different prostate cancer cells was as effective in the inhibition of proliferation as treatment with the Hh-pathway inhibitor cyclopamine.

Hh signaling inhibition also substantially reduced neo-angiogenesis. Xenografts from PC3 prostate cancer cells expressed high levels of SHH, producing small, disorganized vessels typical of tumour vasculature. These results are in line with other reports linking the Hedgehog pathway with the formation of new blood vessels and increases in tumor growth and metastasis [7]. By contrast, tumors treated with hedgehog inhibitors like GDC-0449 and pre-treated with PN1 recombinant protein exhibited altered vasculature with fewer vessels and larger overall diameters. The consequence of reduction in hedgehog signaling was decreased overall vascular density and slower tumor growth.

The capacity to reduce tumor growth and vessel formation suggests a new physiological significance for PN1 and perhaps other serpins with similar functions. Interestingly, PN1 features some similarities to maspin, a tumor suppressor and angiogenesis blocker. Maspin has also been reported to reduce uPA-mediated CaP [8]. However, PN1 is more stable in a purified recombinant state than maspin and may actually have a larger range of inhibitory targets or cell type interactions [8]. For example, a recent report has observed that PN1 may be able to interact with endothelial cells, the initiators of angiogenesis [9].

As a regulatory toggle, we have shown that PN1 is also a target of MMP9-mediated degradation. The localisation of PN1 in the ECM allows for its regulation by MMP9, an important factor in metastasis that can cleave collagen and basement membrane to drive this process. We extended these findings to show that higher MMP9 levels are inverse to PN1 levels and that this ratio is important in controlling hedgehog signaling. We demonstrate competing regulation of hedgehog signaling by PN1 and MMP9 in tissue culture, normal prostate tissues, xenografts, and orthotopic prostate cancers.

**Figure 1:**
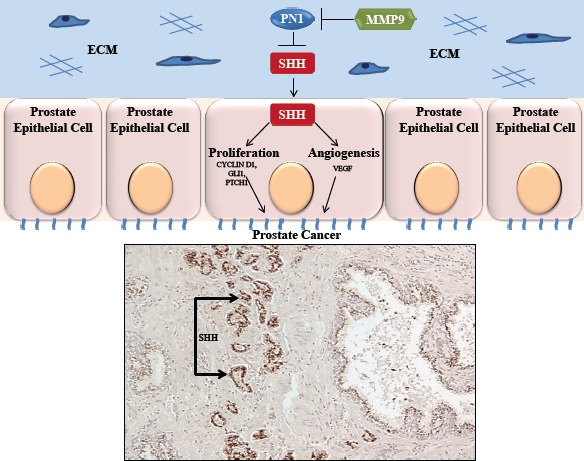
Expression of SHH in prostate cancer Above: PN1- mediated blockade of SHH downstream signaling slows cancer proliferation and neo-angiogenesis. The effect can be inhibited by MMP9. Below: whole mount prostate tissue shows that SHH staining (brown) occurs primarily in prostate tumour cells rather than normal prostate.

The presence of PN1 in normal and BPH (benign prostate hypertrophy) tissues and decline in higher Gleason score samples may indicate that its loss is a marker of malignant transition in the prostate epithelium, amplifying Hh signaling. Indeed, one aim was to utilize Hh signalling as a stratifier of intermediate grade prostate cancer as the transitional status of these patients often makes treatment uncertain. Implications are that pharmacological inhibition of hedgehog signaling might be useful in this subset of patients. Thus, PN1 itself might be a potential therapeutic agent and, in combination with other SHH inhibitors, may be useful in the encouragement of CaP growth lag or inhibition. This work further indicates the potential important of hedgehog signaling in prostate cancer (Figure 1). Given its emerging abilities to affect changes in proliferation, angiogenesis, and invasion, PN1 expression may be a suitable target for improving prostate cancer therapy.
